# High cortactin expression in B-cell acute lymphoblastic leukemia is associated with increased transendothelial migration and bone marrow relapse

**DOI:** 10.1038/s41375-018-0333-4

**Published:** 2018-12-20

**Authors:** Martha Velázquez-Avila, Juan Carlos Balandrán, Dalia Ramírez-Ramírez, Mirella Velázquez-Avila, Antonio Sandoval, Alfonso Felipe-López, Porfirio Nava, José Antonio Alvarado-Moreno, David Dozal, Jessica L. Prieto-Chávez, Matthias Schaks, Klemens Rottner, Elisa Dorantes-Acosta, Briceida López-Martínez, Michael Schnoor, Rosana Pelayo

**Affiliations:** 1 Unidad de Investigación Médica en Enfermedades Oncológicas, UMAE Hospital Oncología, Instituto Mexicano del Seguro Social, Mexico City, Mexico; 2 Centro de Investigación Biomédica de Oriente, Delegación Puebla, Instituto Mexicano del Seguro Social, Metepec, Puebla Mexico; 3 Department of Molecular Biomedicine, CINVESTAV-IPN, 07360 Mexico City, Mexico; 4 Clinica de las Leucemias y Servicios Auxiliares de Diagnóstico, Hospital Infantil de Mexico Federico Gómez, SSA., Mexico City, Mexico; 5 Hospital para el Niño, Instituto Materno Infantil del Estado de México, Toluca, Estado de México Mexico; 6 Department of Physiology, Biophysics and Neurosciences, CINVESTAV, IPN, Mexico City, Mexico; 7 Unidad de Investigación Médica en Trombosis, Hemostasia y Aterogénesis, Instituto Mexicano del Seguro Social, Mexico City, Mexico; 8 Unidad de Investigación Médica en Inmunoquímica, UMAE Hospital de Especialidades, Instituto Mexicano del Seguro Social, Mexico City, Mexico; 9 Division of Molecular Cell Biology, Zoological Institute, TU Braunschweig, 38106 Braunschweig, Germany; 10 Department of Cell Biology, Helmholtz Centre for Infection Research, 38124 Braunschweig, Germany; 11 Present Address: Laboratorio de Biología Molecular y Bioseguridad Nivel III Hospital General Naval de Alta Especialidad 04470 Coyoacán, Ciudad de, Mexico, Mexico

**Keywords:** Acute lymphocytic leukaemia, Cell biology

## Abstract

Cancer is a major cause of death in children worldwide, with B-lineage cell acute lymphoblastic leukemia (B-ALL) being the most frequent childhood malignancy. Relapse, treatment failure and organ infiltration worsen the prognosis, warranting a better understanding of the implicated mechanisms. Cortactin is an actin-binding protein involved in cell adhesion and migration that is overexpressed in many solid tumors and in adult B-cell chronic lymphocytic leukemia. Here, we investigated cortactin expression and potential impact on infiltration and disease prognosis in childhood B-ALL. B-ALL cell lines and precursor cells from bone marrow (BM) and cerebrospinal fluid (CSF) of B-ALL patients indeed overexpressed cortactin. In CXCL12-induced transendothelial migration assays, transmigrated B-ALL cells had highest cortactin expression. In xenotransplantation models, only cortactin^high^-leukemic cells infiltrated lungs, brain, and testis; and they colonized more easily hypoxic BM organoids. Importantly, cortactin-depleted B-ALL cells were significantly less efficient in transendothelial migration, organ infiltration and BM colonization. Clinical data highlighted a significant correlation between high cortactin levels and BM relapse in drug-resistant high-risk B-ALL patients. Our results emphasize the importance of cortactin in B-ALL organ infiltration and BM relapse and its potential as diagnostic tool to identify high-risk patients and optimize their treatments.

## Introduction

Childhood cancer is a global health priority [1–3], with leukemia remaining a leading cause of morbidity and mortality in children worldwide, showing incidence rates of 140.6 per million new cases per year in ages of 0–14 [1]. Among leukemias, B-precursor cell acute lymphoblastic leukemia (B-ALL) represents 73–85% of total cases [4]. Current therapies have increased overall survival rates up to 80%. However, organ infiltration and relapse are still correlated with poor prognosis [5], warranting the search for more accurate diagnostic tools to identify high-risk groups. Bone marrow (BM) relapse is most common and clinically complicated, but infiltration to extramedullary tissues such as central nervous system (CNS) also occur and are related to relapse [5–7]. Factors promoting cell adhesion, transendothelial migration (TEM), and homing may trigger organ infiltration [8, 9]. Adhesion and migration is regulated by the actin cytoskeleton via formation of protrusive structures and clustering of adhesion molecules to allow for B-cell interaction with stromal cells in the BM or with vascular endothelial cells. Cortactin and its homolog hematopoietic cell-specific lyn substrate 1 (HS1) are actin-binding proteins (ABP) facilitating cell adhesion and migration [10]. Cortactin is upregulated in several cancers to trigger cell migration and invasiveness [11], and both cortactin and HS1 are related to poor prognosis in adult B-cell chronic lymphocytic leukemia (B-CLL) [12–15], and linked to high levels of the known risk factors ZAP70 and CD38 [16]. Cortactin also participates in the internalization and trafficking of the CXCL12-receptor CXCR4 [17, 18]. Of note, CXCL12 is constitutively produced in BM and CNS to regulate homing, adhesion and TEM of B-progenitors mediated by the integrins VLA-4 and LFA-1 [19, 20]. Thus, we hypothesized that cortactin triggers the transmigratory capacity of leukemic B-ALL cells in children. We show that leukemic B-ALL cell lines and primary pediatric B-ALL cells express high levels of cortactin that are required for TEM and organ infiltration in vitro and in vivo. We also provide clinical evidence that high cortactin levels in B-ALL correlate with BM relapse.

## Materials and methods

### Patients

BM aspiration and CSF samples were collected according to international and institutional guidelines from children and adolescents younger than 18 years and diagnosed with B-ALL before any treatment or upon relapse. All samples were collected after written informed consent from parents. Patients were recruited and followed in the Hospital Infantil de Mexico “Federico Gomez” (Mexico City) and the IMIEM Hospital para el Niño (Toluca, Mexico) (all available clinical information is summarized in Suppl. Table 1). All procedures were approved by the Ethics, Research and Biosafety Committees of the hospitals and CINVESTAV.

### Cell culture

Cell lines REH and RS4:11 and stromal HS-5 and OP9 cells were obtained from ATCC, were free of mycoplasma, and cultured according to the provided protocols. Stable cortactin-depleted REH cells were generated by lentiviral infection using pLentiCRISPRv2 vector (Plasmid #52961, Addgene, Cambridge, MA), and the following gRNA sequences: CTTN-2 ATCGGCCCCCGCGTCATCCT; and CTTN-3 GTCCATCGCCCAGGATGACG. These gRNAs reduced cortactin expression, but CTTN-3 resulted in highest reduction of around 40% (Suppl. Figure 1); thus, these cells were used for all functional experiments. Human umbilical vein endothelial cells (HUVEC) were cultured in EGM-2 medium (Lonza, Switzerland) with 10% FBS. Mononuclear cells from BM specimens from 23 pediatric patients (Suppl. Table 1) were purified by Ficoll-Paque (GE Healthcare) and the CD34^+^-fraction enriched using the human CD34 MicroBead-Kit-UltraPure (MiltenyiBiotec, Germany). Progenitor cells were identified as Lineage^−^CD34^+^, Pro B cells as CD34^+^CD19^+^, and Pre B as CD34^−^CD19^+^. Mononuclear cells from umbilical cord blood (UCB) were used as control. CSF was collected after lumbar puncture, cytospinned and fixed with 3% (paraformaldehyde) PFA.

### Quantitative RT-PCR

Expression of cortactin and HS1 was analyzed by real time RT-PCR, using the following primers: Cortactin 5′-ggtgtgcagacagacagacaa-3′ and 5′-gtctttttgggattcatgcag-3′; HS1 5′-cccaagagtcctctctatcctg-3′ and 5′-ggtggcagagaggtgttcat-3′; β2-microglobulin (housekeeping gene) 5′-tcaggaaatttgactttccattc-3′ and 5′-ttctggcctggaggctatc-3′. RNA from REH cells and UCB CD34^−^CD19^+^ populations was obtained using TRIzol (Thermo Fisher). cDNA was synthetized from 1.5 µg of total RNA using SuperScript II (Thermo Fisher) and amplified using these conditions: pre-incubation: 95 °C, 10 min; amplification: 40 cycles of 95 °C, 10 s; 60 °C, 30 s; 72 °C, 10 s. Relative gene expression was calculated using the ΔΔCt-method.

### Flow cytometry

Cells were stained in PBS with 3% FBS for 20 min using the following antibodies (Biolegend): anti-hCD34-PB (#343512), anti-hCD19-APC (#302212), anti-hCD19-PE (#302208), anti-hCD184-PE-Cy7 (#306514), anti-hCD62L-PE (#304805), anti-hCD162-APC (#328805), anti-hCD11b-APC (#301309), anti-hCD18-PE (#373407), anti-hCD11a-PE (#301207), anti-hCD18-APC (#373405), anti-hCD49d-PE (#304303), anti-hCD29-APC (#303007), and anti-hCXCR4-APC (Abcam, #124828). Cortactin and HS1 expression was evaluated after permeabilization and fixation with cytofix/cytoperm (BD Bioscience) for 20 min at 4 °C, before incubation for 30 min with anti-cortactin-Alexa488 clone 289H10 [21], or with anti-HS1 (D83A8; Cell Signaling) and goat anti-rabbit-Alexa488. Cells were analyzed using a FACSCantoII and FlowJo V10.4.2. MNCs from ALL BM or UCB were stained with anti-hCD34-PE-Cyanin5 (BD) and anti-hCD19  APC (Biolegend) and sorted in a BD FACS Aria II.

### Immunofluorescence microscopy

Cells were fixed with 3% PFA, permeabilized with 0.1% Triton X-100 and blocked with 5% BSA in PBS for 1 h, followed by incubation with primary antibodies overnight at 4 °C. After washing, cells were incubated with Alexa-Fluor-labelled secondary antibodies and mounted in Vecta-Shield containing DAPI. Samples were analyzed using a confocal laser scanning microscope (Leica TCS-SP8).

### Western blotting

B-ALL cells were lysed in 25 mM HEPES, pH 7.5, 150 mM NaCl, 10 mM MgCl_2_, 1 mM EDTA, 1% NP-40, 2% glycerol with protease inhibitors; and analyzed by standard protocols using anti-cortactin [21] and anti-γ tubulin (Sigma, #T6557) antibodies.

### Transendothelial migration

HUVEC were grown to confluence on 5 µm-pore transwell-filters (Corning) pre-treated with attachment factor (Gibco, Thermo Scientific). A total of 1.5 × 10^4^ REH, RS4:11 or MNCs were resuspended in 200 µl EGM-2 medium and placed on the endothelial monolayer followed by addition of 100 ng/ml CXCL12 (Peprotech) to the bottom. In some cases, HUVEC were pre-activated with 50 nM TNFα for 16 h. After 4 h, transmigrated cells from the bottom chamber were counted and cells from the top and bottom chambers stained with anti-cortactin and analyzed by flow cytometry. For competition assays, REH and RS4:11 cells were pre-labeled with Celltrace-far-red and Celltrace-violet (Thermo Fisher).

### B-ALL-stromal cell co-culture

HS-5 human stromal cells were plated at a density of 1 × 10^5^ cells per coverslip. After 24 h, REH cells were stained with Celltrace-violet (Thermo Fisher) and 3 × 10^5^ cells placed on top of the stromal cell monolayer. Co-cultures were fixed after 24 h with 3% PFA and stained using anti-cortactin and anti-CXCR4 antibodies.

### Tridimensional co-culture system

Mesenchymal stromal OP9 cells were induced to form spheroids in non-adherent conditions, and B-ALL cell lines or leukemic precursors from patients were co-cultured with spheroids as described [22]. Spheroids were removed from co-cultures and washed several times with PBS-EDTA (0.05 mM) to detach surface cells. Then spheroids were trypsinized and mechanically disrupted. Flow cytometry analysis was performed after immunostaining of cortactin and hCD45 in cells of the digested spheroid (IN), as well as in cells collected from the culture supernatant after spheroid removal (OUT). Once spheroids have formed they are stable, and do not continuously break down and reform in cultures. For competition assays, B-ALL cells were pre-labeled as above.

### Cell cycle analysis

Cells were collected from 3D co-cultures or from BM of transplanted NSG mice, and stained with anti-hCD45 and anti-mCD45. After fixation and permeabilization, Ki67 and cortactin were stained together with DAPI and analyzed by flow cytometry.

### Xenografts

All animal studies were approved by the IACUC of CINVESTAV, and performed according to the ethical guidelines established by the Mexican authorities. A total of 3 × 10^6^ REH cells were injected via the tail vein into non-irradiated male NSG mice (Jackson Laboratories, ME, USA). At least 3 mice per group in each independent experiment were used. Mice were kept in the animal facility of CINVESTAV and leukemia progression was monitored weekly by flow cytometry of peripheral blood. Once disease was established, mice were killed and BM, liver, spleen, brain, lung, and testis were collected, digested, and analyzed by flow cytometry.

### Statistics

Prism V3.02 (GraphPad) software was used. Multivariant statistical analysis was performed to correlate cortactin levels with CNS infiltration or BM relapse. Differences within groups were established by non-parametric tests, considering probability values <0.05 as statistically significant. Mann–Whitney *U*-test was applied for clinical data. Data were normally distributed, and variances were similar between the compared groups.

## Results

### The 60 kDa cortactin SV2 isoform is expressed in B-ALL cells and determines their transmigratory capacity

**Fig. 1:**
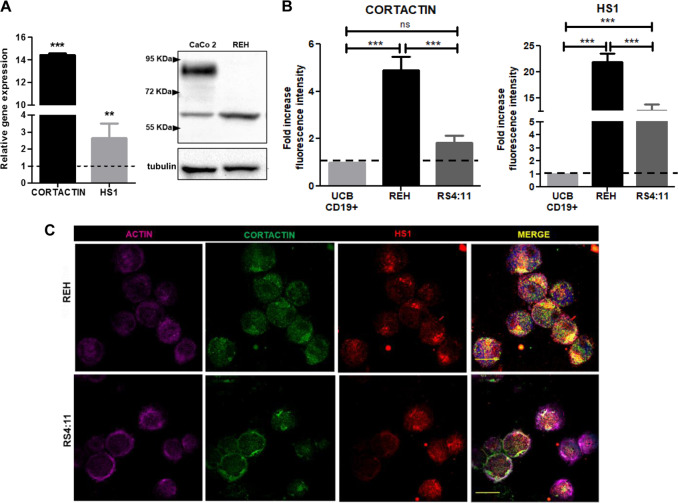
B-ALL cell lines express the cortactin SV2 isoform. **a** qRT-PCR for cortactin and HS1 was performed with cDNA from REH cells or umbilical cord blood (UCB)-derived CD19^+^-cells as control (left panel). Data were analyzed by the ΔΔCt-method and are depicted as fold-change expression relative to control cells (set to 1, dotted line) normalized to the housekeeping gene β-2-microglobulin (*n* = 3). Western blots revealed that REH cells express the SV2 isoform of 60 kDa (right panel). CaCo-2 epithelial cells served as positive control for wild-type cortactin (80 kDa) (*n* = 3). **b** Flow cytometry of cortactin and HS1 protein levels in REH and RS4:11 cells normalized to cortactin and HS1 expression in UCB-derived CD19^+^ cells as controls (dotted lines). Data are displayed as fold-increase fluorescence intensity (*n* = 3). **c** Representative immunofluorescence stainings of REH and RS4:11 cells for cortactin, HS1 and actin (*n* = 2). Bar = 20 µm

First, we analyzed the mRNA levels of the ABP cortactin and HS1 in the Pre B-cell line REH isolated from a B-ALL relapse patient. HS1 is the hematopoietic cell-specific cortactin homolog. It is actually not known, whether the two proteins interact with each other or show functional redundancy as until recently they were thought to be expressed in different cells. However, both HS1 and cortactin mRNAs were significantly overexpressed in REH cells when compared to non-malignant UCB-CD19^+^-cells (Fig. 1a, left). Whereas HS1 expression was almost three times higher compared to control cells, cortactin was increased 14-fold in REH cells compared to control cells. Western blot analysis of whole-cell lysates revealed that in REH cells the 60 kDa splicing variant 2 (SV2) isoform of cortactin was present, in contrast to the epithelial cell line CaCo-2 that predominantly expresses the 80 kDa cortactin WT isoform (Fig. 1a, right). Quantification revealed a significant 2.9-fold higher expression of the 60 kDa band in REH cells compared to CaCo-2 cells. The 60 kDa isoform results from alternative splicing and lacks the last 2.5 repeats within the actin filament (F-actin)-binding region, suggesting altered affinities for F-actin [23]. However, the functional relevance of differential isoform expression remains elusive. IF stainings for cortactin and HS1 in REH cells showed presence of both proteins within the cytosol and at the cell periphery (Suppl. Figure 2). Flow cytometry analysis confirmed significantly higher expression of cortactin in REH cells, whereas there was only a tendency towards higher cortactin levels in the B-ALL cell line RS4:11 compared to UCB CD19^+^ cells (Fig. 1b, left). Both cell lines were established from relapsed patients, with the difference that REH derived from peripheral blood, and RS4:11 from bone marrow. We exploited this difference in further experiments as cortactin^high^ (REH) and cortactin^low^ (RS4:11). HS1 expression was higher in both B-ALL cell lines (Fig. 1b, right). Both proteins localized in proximity to actin in REH and RS4:11 cells (Fig. 1c).

**Fig. 2:**
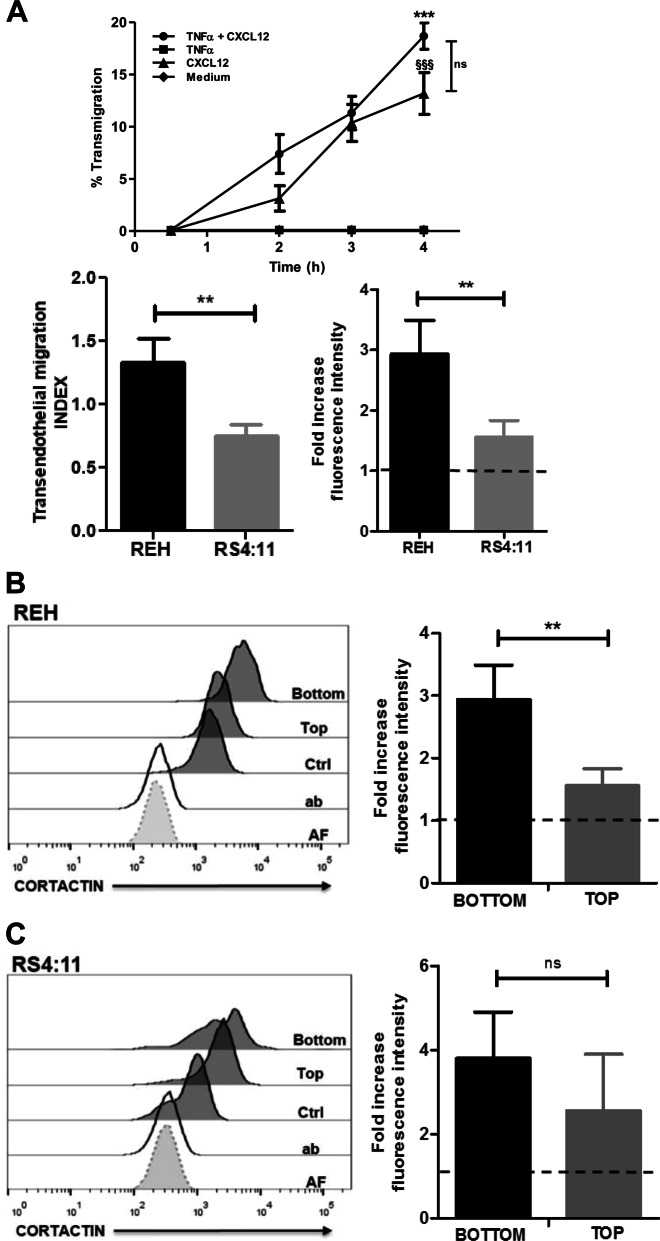
High cortactin levels increase the transmigratory capacity of B-ALL cells in response to CXCL12. **a** In transendothelial migration assays, REH cells transmigrate in a CXCL12-dependent fashion, but do not require endothelial pre-activation by TNFα (left panel). Statistical analysis of transmigration kinetics was performed by one-way ANOVA with Bonfferoni’s post hoc test. * indicates TNFα + CXCL12 vs TNFα; § indicates CXCL12 vs medium; *p* < 0.001. Percentage of transmigrated cells after 4 h was calculated from the proportion of cells in the bottom (transmigrated cells) and top (non-transmigrated cells) (*n* = 3). TEM competition assays with labeled REH and RS4:11 cells in a 1:1 ratio (middle panel). The TEM index was calculated from cell numbers of each cell line in the bottom and showed that REH cells transmigrated more efficiently (*n* = 3). Transmigrated REH cells express more cortactin (right panel, *n* = 3). Fold increase values are normalized to cortactin expression in UCB-derived CD19^+^ cells (dotted line). **b**, **c** Cortactin levels in transmigrated (bottom) REH **b** and RS4:11 **c** cells were higher than in non-transmigrated (top). Data are displayed as fold increase of mean fluorescence intensity normalized to the mean fluorescence intensity of UCB-MNC control cells that were not subjected to transmigration (*n* = 3). **p < 0.01; ns non-significant

As cortactin regulates leukocyte extravasation [24], we wondered whether high cortactin levels provide an advantage during TEM. We found that 20% of REH cells transmigrated across HUVEC monolayers in response to CXCL12 (Fig. 2a). Interestingly, transmigration was independent of endothelial pre-activation with TNFα. Competitive transmigration assays using 1:1 cell suspensions of cortactin^high^-REH and cortactin^low^-RS4:11 cells showed that REH transmigrated significantly more than RS4:11 (Fig. 2a, lower left), and expressed significantly more cortactin as determined by flow cytometry (Fig. 2a, lower right). Analyzing cortactin abundance in transmigrated cells recovered from the bottom chambers vs non-transmigrated cells from the top of the endothelial monolayer revealed that transmigrated REH cells had significantly higher levels of cortactin compared to non-transmigrated cells (Fig. 2b), while transmigrated RS4:11 only showed a tendency towards higher levels when compared to non-transmigrated cells (Fig. 2c).

Leukocyte transmigration depends on selectins, integrins, and chemokine receptors [25]. However, although we observed substantial surface expression of the VLA-4 subunits CD49d and CD29, expression of L-selectin, PSGL1, Mac-1, and LFA-1 was rather low and not increased by CXCL12 (Suppl. Figure 3). Both cell types had significant surface amounts of CXCR4, which decreased after CXCL12 treatment, probably due to internalization [26].

### Infiltrating and relapse cells from pediatric B-ALL patients show highest levels of cortactin

Whether a subset of malignant B-cell precursors is particularly invasive is still a matter of debate. Flow cytometry analyses of sorted patient-derived primary BM B-ALL cell subpopulations, including CD34^+^CD19^−^-progenitors, CD34^+^CD19^+^-Pro B and CD34^−^CD19^+^-stages, showed heterogeneous cortactin expression among patients and lineage differentiation (Suppl. Figure 4A). Nevertheless, IF data suggested that cortactin levels decreased during maturation; whereas, HS1 remained constant (Suppl. Figure 4B).

**Fig. 3:**
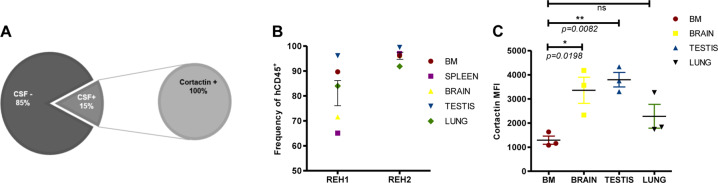
High cortactin expression levels in early leukemic populations from B-ALL patients are associated with secondary organ infiltration. **a** Cerebrospinal fluids (CSF) were positive for infiltrated leukemic cells in 15% of B-ALL patients (*n* = 26), and 100% of infiltrated B-cells expressed cortactin. **b**, **c** NSG mice were xeno-transplanted with REH or primary B-ALL cells. REH-derived xenografts (REH-DX) and patient-derived xenografts (PDX) were analyzed for infiltrated cells in BM, brain, testis, and lungs by flow cytometry. **b** Cell frequencies of human hematopoietic cells (hCD45^+^) from total leukocyte counts (mCD45^+^ and hCD45^+^) were recorded 5 weeks (REH1) or 6 weeks (REH2) after transplantation (left panel). Representative plots of gated live, infiltrated human leukocytes are shown in Suppl. Figure 5B for REH-DX and PDX. **c** Cortactin levels in hCD45^+^-cells derived from BM, brain, testis, and lungs analyzed by flow cytometry (left panel, *n* = 3). Cortactin values of each xeno-transplant in the tested tissues are shown in Suppl. Figure 5C

Given the roles of these ABP in cell adhesion and migration, we speculated that they could be linked to disease relapse often involving secondary organ infiltration. Thus, we collected CSF from 26 B-ALL patients and discovered that 15% of these samples were positive for cellular infiltration, and that 100% of infiltrated leukemic cells expressed cortactin and HS1 (Fig. 3a and Suppl. Figure 5A). The role of cortactin in in vivo migration and extramedullary infiltration was explored in REH- and patient-derived leukemia xenografts (REH-DX and PDX, respectively). Once the xeno-transplanted NSG mice exhibited leukemic burden or disease signs, they were killed and BM, brain, testis, lung, liver, and spleen processed and analyzed for infiltration by human CD45^+^-hematopoietic cells (Fig. 3b and Suppl. Figure 5B). Both, REH-DX and PDX preclinical models demonstrated leukemic cell infiltration of brain, testis, and lung, organs known to be targets for leukemic infiltration [6, 27]. Importantly, infiltrated cells expressed cortactin, with the highest levels detected in B-ALL cells isolated from brain, testis, and lung (Fig. 3c and Suppl. Figure 5C), strongly suggesting that only B-ALL cells with high cortactin levels are capable of inducing infiltrative B-ALL disease in the PDX model.

**Fig. 4:**
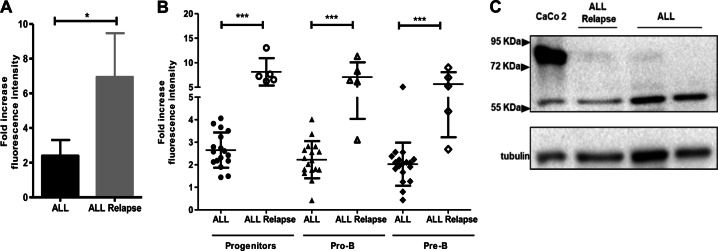
The cortactin SV2 isoform is expressed in B-ALL precursors. **a** Cortactin expression was investigated by flow cytometry in progenitor and precursor B-cells from BM of children diagnosed with either B-ALL (*n* = 18) or B-ALL with BM relapse (*n* = 5). **b** Cortactin levels in progenitors, pro-B and pre-B cells from B-ALL patients at debut or BM relapse. Data in **a** and **b** are presented as fold increase of mean fluorescence intensity normalized to UCB-MNC. *p < 0.05; ***p < 0.001. **c** Western blot of total peripheral blood MNC from newly diagnosed and relapsed B-ALL patients showed that they express the 60 kDa SV2 isoform. CaCo-2 cells mostly express wild-type cortactin (80 kDa) (*n* = 3)

Surprisingly, cortactin levels in B-ALL cells from patients with BM relapse were increased threefold compared to cells from newly diagnosed patients (Fig. 4a, Table 1), and this was true in all cell subsets (Fig. 4b). Of note, statistical analyses to define whether cortactin levels relate to particular clinical disease features confirmed a significant correlation with leukemic relapse to BM, steroid treatment failure, and adenomegaly (Table 1). However, cortactin overexpression was not related to sex, risk, immunophenotype, infections, cytopenia, hepatomegaly, or splenomegaly (Table 1). Pearson’s correlation tests also identified increased platelet numbers as significant correlation (Suppl. Figure 6); however, body-mass index, LDH, hemoglobin levels, numbers of leukocytes and blasts in PB and BM were not significantly correlated to cortactin overexpression (Suppl. Figure 6). Like in non-relapsed B-ALL cells, the SV2 cortactin isoform was expressed in cells from relapsed patients (Fig. 4c).

**Table 1:** Cortactin expression levels among clinical variable groups of B-ALL patients (Mann–Whitney *U*-test with an α of 5% was applied to define statistical significance in bolded P- and Fold increase- values)

Variable	*P*-value		Fold increase of mean fluorescence intensity of cortactin expression
Sex	0.137	Female (*n* = 10)	2.62
		Male (*n* = 13)	2.46
Risk	0.382	High (*n* = 14)	4.13
		Standard (*n* = 3)	2.08
Hyperleukocytosis	0.999	Positive (*n* = 4)	3.17
		Negative (*n* = 14)	3.61
Immunophenotype	0.815	Pro B; Pro/Pre B (*n* = 16)	3.65
		Pre B (*n* = 7)	2.96
Translocation	0.556	Positive (*n* = 6)	3.50
		Negative (*n* = 9)	3.71
Response to steroids	**0.051**	Positive (*n* = 13)	**2.86**
		Negative (*n* = 3)	**6.57**
Infections	0.545	Positive (*n* = 3)	3.01
		Negative (*n* = 13)	3.68
Cytopenia	0.533	Positive (*n* = 10)	3.47
		Negative (*n* = 3)	3.74
Hepatomegaly	0.711	Positive (*n* = 9)	3.24
		Negative (*n* = 7)	3.97
Splenomegaly	0.903	Positive (*n* = 12)	3.68
		Negative (*n* = 4)	3.52
Adenomegaly	**0.013**	Positive (*n* = 13)	**4.03**
		Negative (*n* = 13)	**1.49**
Relapse	**0.001**	Positive (*n* = 5)	**6.94**
		Negative (*n* = 18)	**2.37**
Death	0.313	Positive (*n* = 3)	4.31
		Negative (*n* = 13)	3.38

Treatment of REH cells with the chemotherapeutics vincristine, prednisolone, and dexamethasone revealed that cortactin expression was not significantly affected suggesting that during remission-inducing treatment residual cells do not alter cortactin expression as an associated rebounding mechanism, or due to a direct effect on transcription (Suppl. Figure 7). Thus, subsequent relapse may not result from selection of cortactin^high^ malignant clones induced by chemotherapy, but from highly migratory clones with high intrinsic cortactin expression.

### Cortactin^high^-leukemic cells are endowed with HSC niche-positioning and cycling properties

**Fig. 5:**
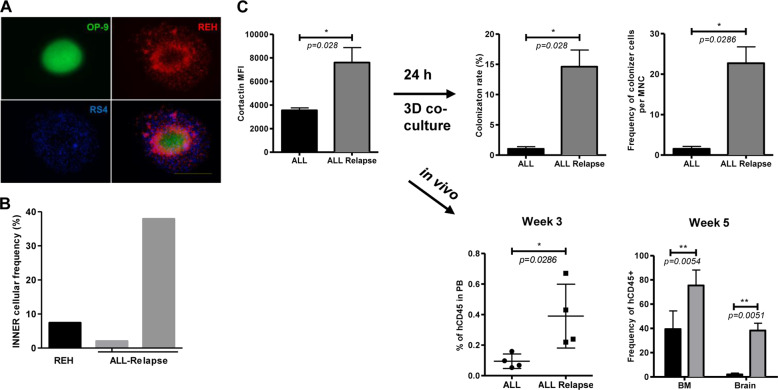
Cortactin levels correlate with bone marrow colonization potential, bone marrow engraftment, and organ infiltration of relapsed B-ALL cells. **a** Representative image of labeled RS4:11 and REH cell lines co-colonizing a 3D-BM stromal spheroid (*n* = 3). Bar = 400 µm. **b** The potential for BM colonization of REH and primary relapsed B-ALL cells from two different patients were quantified from 3D-BM stromal spheroid co-cultures (compare also Suppl. Figure 8). Frequencies of hCD45^+^-cells that colonized the spheroids (percentage of total applied cells). **c** Primary B-ALL cells from newly diagnosed (ALL) and relapse patients (ALL-Relapse) with distinct levels of cortactin (left panel) were tested in BM-spheroid colonization (right panel), in which ALL-Relapse cells colonized the spheroids significantly more efficient. In xenotransplantation assays ALL-Relapse cells established a more severe disease (lower left panel), and infiltrated BM and brain more efficiently (lower right panel). Analyses were performed by flow cytometry after human CD45 staining. *n* = 3 for all experiments; *p < 0.05; **p < 0.01

Given the possible temporal and spatial co-existence of normal, non-relapse and relapse B-ALL cells within the same internal hematopoietic zones of leukemic maintenance, we tested the ability of cortactin^high^-relapse B-ALL cells to home into central hypoxic niches. Competition colonization assays using REH and RS4:11 cells in a spheroid-like tridimensional stromal cell co-culture system [22] showed a clear advantage of cortactin^high^-REH cells in entering the spheroid (Fig. 5a). Importantly, only cells expressing the highest amounts of cortactin of both REH and primary B-ALL cells could efficiently colonize the stromal cell spheroid. Non-colonizing B-ALL cells from the cell suspension surrounding the spheroid had much lower cortactin levels (Suppl. Fig. 8). However, cell frequencies (percentage of cells within spheroids with respect to total number of applied cells) varied among REH and primary cells (Fig. 5b).

Moreover, we performed BM spheroid colonization experiments and xenotransplantation experiments in NSG mice using primary cells from B-ALL patients at debut and relapse. Interestingly, B-ALL relapse cells that expressed significantly higher cortactin levels colonized BM spheroids more efficiently, caused a more severe disease in NSG mice and infiltrated bone marrow and brain stronger than B-ALL debut cells (Fig. 5c). However, it will require further analysis to clearly state whether this is a correlative or causative effect.

Establishment of pre-leukemic cells in lymphoid BM niches is CXCL12-dependent (22). Thus, we wondered whether cortactin participates in niche stabilization via CXCR4, and observed discrete proximity of CXCR4 and cortactin staining at cell contacts of B-ALL and stromal cells (Suppl. Fig. 9A). Moreover, a tendency toward higher surface CXCR4 expression was apparent in B-ALL cells from BM samples of relapsed patients, compared to BM samples of newly diagnosed patients (Suppl. Fig. 9B). Vice versa, we found that basal surface CXCR4 levels were reduced in cortactin-depleted REH cells (Suppl. Fig. 10A), whereas basal total CXCR4 levels were not significantly changed (Suppl. Fig. 10B). Of note, treatment with CXCL12 resulted in similar CXCR4 internalization in both control and cortactin-depleted cells (Suppl. Fig. 10A), suggesting that in B-ALL cells cortactin is important for basal CXCR4 cell surface localization, but not for CXCL12-dependent CXCR4 internalization as described in T-ALL cells [18]. Given that basal CXCR4 surface levels are lower without cortactin, it is likely that cortactin affects transmigration towards CXCL12 via CXCR4. All these data suggest that transendothelial migration and BM colonization by cortactin^high^-B-ALL cells is an important event during relapse.

**Fig. 6:**
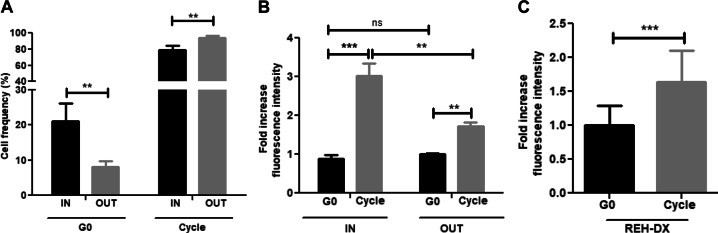
Cortactin^high^-B-ALL cells that home to BM niches are in cell cycle. Stromal spheroids were co-cultured with REH cells for 24 h, followed by flow cytometry analyses to identify whether leukemic cells within spheroids (IN) or in suspension (OUT) are in cell cycle. **a** Cell cycle status was determined by Ki67 and DAPI staining, and cell frequencies of quiescent (G0) and cycling (G1-S-G2-M) IN and OUT populations were calculated. **b** Cortactin levels in quiescent (G0) and cycling (G1-S-G2-M) cells were investigated by flow cytometry and normalized to values of G0 cells (IN) in CD45^+^-cells (*n* = 3). **c** NSG mice were xeno-transplanted with REH cells. Upon disease establishment, mice were killed and REH-derived xenograft (REH-DX) cells collected from BM and stained with anti-mCD45, anti-hCD45, anti-hKi67, and DAPI. Cortactin levels in G0 and cycling REH-DX BM cells were determined by flow cytometry and normalized to G0 cells (IN) (left panel, *n* = 2). **p < 0.01; ***p < 0.001

Normal cells residing in hematopoietic stem cell niches within BM are mostly quiescent and intermittently enter the cell cycle. Thus, we investigated whether this is also true for leukemic precursors at disease relapse by using our organoid-like tridimensional co-culture system [22]. This system provides natural microenvironments like those constituting lymphoid BM reticular niches, including stromal interconnection and internal CXCL12^+^-hypoxic zones. We recovered significantly more G_0_ cells from within the spheroids (Fig. 6a). However, the highest cortactin expression was recorded in the cycling cell population that entered the spheroids, whereas cortactin was lowest in the quiescent populations (Fig. 6b and Suppl. Fig. 11). The REH-DX model confirmed that cortactin^high^-leukemic cells in the BM were cycling in vivo (Fig. 6c). Thus, our study clearly shows that cortactin^high^-B-ALL progenitors are mainly migrating and proliferating cells endowed with the capability of colonizing hypoxic hematopoietic internal zones of leukemic maintenance and infiltrating extramedullary organs.

### Cortactin depletion in REH B-ALL cells reduces their ability to transmigrate, colonize BM spheroids, and infiltrate organs

**Fig. 7:**
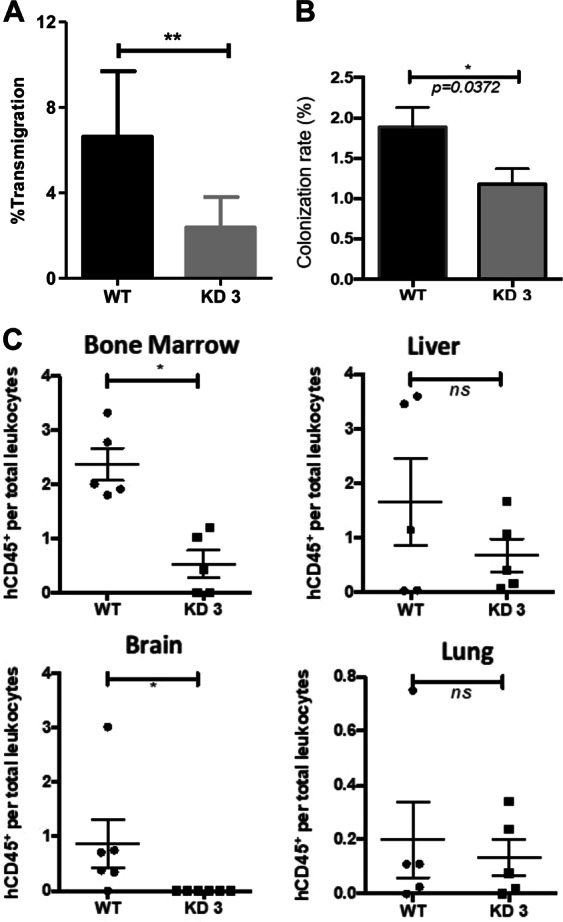
Cortactin is required for competent leukemic cell migration, bone marrow engraftment, and organ infiltration. **a** Transendothelial migration assays were performed with cortactin knockdown 3 (KD3) REH and control cells across confluent HUVEC monolayers towards CXCL12 gradients. The amount of transmigrated B-ALL cells is shown as percentage of total applied cells. **b** Stromal spheroids were co-cultured with labeled WT or KD3 REH cells and composition of spheroids after 24 h was determined by flow cytometry. **c** Xenotransplantation assays were performed by i.v. injection of WT or KD3 REH cells into NSG mice. WT REH cells showed 100% bone marrow (BM) engraftment efficiency; whereas, the KD cells established the disease in BM in only 3 of 5 mice (60% of engraftment efficiency). Overall engraftment and infiltration of organs by leukemic cells was investigated by flow cytometry of hCD45 vs mCD45 staining. *n* = 3 for all experiments; *p < 0.05; **p < 0.01; ns not significant

In order to strengthen the idea that cortactin is indeed required for providing the transmigratory advantage to cortactin^high^ B-ALL REH cells, we generated stable cortactin-depleted REH cells (CTTN-KD-3) using CRISPR/Cas9 technology. Transwell-filter transmigration across CXCL12-stimulated HUVEC resulted in a 64% reduction in transmigration of CTTN-KD-3 REH compared to controls (Fig. 7a). Cortactin-depleted REH cells were also less efficient in colonizing BM spheroids (Fig. 7b and Suppl. Fig. 12A). Importantly, xenotransplantation experiments in NSG mice revealed that CTTN-KD-3 cells showed reduced ability to infiltrate organs (Fig. 7c and Suppl. Fig. 12B). Of note, the disease was established in all mice injected with control REH cells, whereas only 3 out of 5 mice injected with CTTN-KD-3 cells established ALL clinical signs. In these mice, CTTN-KD-3 cells infiltrated brain and BM significantly less; whereas a strong (but insignificant) tendency to reduced infiltration in the liver was observed, but no differences in lungs (Fig. 7c). Interestingly, all functional effects were within the range of the observed KD efficiency, thus correlating well with cortactin expression levels. These data corroborate the importance of cortactin overexpression for the migratory advantage and aggressiveness of B-ALL cells.

## Discussion

Cortactin is an ABP relevant for cell functions, such as adhesion and migration, and its overexpression correlates with poor prognosis in many solid cancers [10, 11]. Aberrant cortactin expression was recently detected in the adult B-lineage pathology CLL [16]. Here, we report overexpression of cortactin in B-ALL cells, where it co-localizes with its homolog HS1 and F-actin, implying a role in actin remodeling during formation of lamellipodia and invadopodia as described for other cell types [28–30]. Of note, B-ALL cells at debut and relapse also express the 60 kDa cortactin SV2 variant, in which the fifth and sixth repeats of the F-actin-binding region are deleted [23]. As only the fourth repeat is essential for F-actin-binding, which is conserved in SV2, cortactin in B-ALL cells should be functional, albeit with altered F-actin affinity [31].

Cortactin is important in endothelial cells for ICAM-1 clustering and the regulation of actomyosin contractility [24, 32], and HS1 in leukocytes is important for integrin activation and proper TEM [33]. Accordingly, TEM assays with B-ALL cells confirmed that high cortactin expression conveyed a migratory advantage. Whether this results from a pre-selection of highly migrating cortactin^high^-cells within the leukemic population or from cell activation by environmental signals and subsequently induced cortactin expression needs to be unraveled in future studies. In a number of tumor cells, cortactin activation seems to be stimulated by CXCL12, promoting lamellipodia and invadopodia formation [18, 34, 35]. Vice versa, cortactin also regulates the dynamics of the G-protein-coupled CXCL12-receptor CXCR4 in a calcineurin-dependent fashion [18, 19]. As TEM is a prerequisite for infiltration and cortactin is required for proper TEM, high cortactin expression was also detected in infiltrated B-ALL cells from CSF specimens, and in primary B-ALL cells from xenografted lung, brain, and testis.

Analyzing samples from relapsed patients, we found a positive correlation of high cortactin levels with relapse status, adenomegaly and failure of steroid therapy. Although leukemia relapse is poorly understood, it has been associated with survival of a unique type of leukemia-initiating cell (LIC) with special biological attributes, including resistance to drug therapy that may re-emerge promoted by the microenvironment, and intrinsic genetic differences from that shown by similar clones at first diagnosis [5, 36]. LICs might remain hidden and adhered in specific survival niches located in inner BM structures where therapy does not reach [36, 37]. Under homeostatic conditions, CXCL12 production by BM stromal cells is critical for development of lymphopoiesis, and LIC are potentially established in normal niches coexisting with non-leukemic cells before the blast-driven microenvironmental remodeling that then diminishes CXCL12 and alters normal hematopoiesis [38, 39]. We have recently demonstrated that under pro-inflammatory conditions in the BM, CXCL12 is reduced and HSC hypoxic niches are diluted, allowing gradual leukemogenesis and maintenance of tumor clones [22]. Nevertheless, after therapy and during remission, cellular contents, and biological structures of the BM niches may resemble normality, as CXCL12 production gradually tends to normal values and normal hematopoiesis is re-established. Our data from 3D co-cultures now indicate that non-highly proliferative but cycling relapse cells colonize inner areas in a cortactin-dependent manner, representing hematopoietic zones of leukemic maintenance. We show for the first time that the SV2 variant of cortactin is involved at leukemia debut by providing migratory advantages that are enhanced at relapse where cortactin expression is highest in cells endowed with niche-positioning and cycling properties (Suppl. Fig. 13).

Our findings highlight the importance of cortactin as diagnostic tool for drug-resistant, high-risk patients, and BM relapse. Whether these migrating relapse cells are prompted to re-emerge from such protective/survival niches to cause relapse needs to be investigated. It will also be important to unravel whether pharmacological manipulation of cortactin functions, e.g. by kinase or acetylase inhibitors, could prevent TEM, tissue invasion, and disease relapse.

## Supplementary information

Supplemental Material
