# Supplementary material for: High cortactin expression in B-cell acute lymphoblastic leukemia is associated with increased transendothelial migration and bone marrow relapse
Source: Leukemia. 2018 Dec 20;33(6):1337–48. doi: 10.1038/s41375-018-0333-4 (PMC6756064; doi:10.1038/s41375-018-0333-4)
Supplement: Supplementary file 1 — Supplemental Material [file 41375_2018_333_MOESM1_ESM.pdf]

Supplemental Figure 1

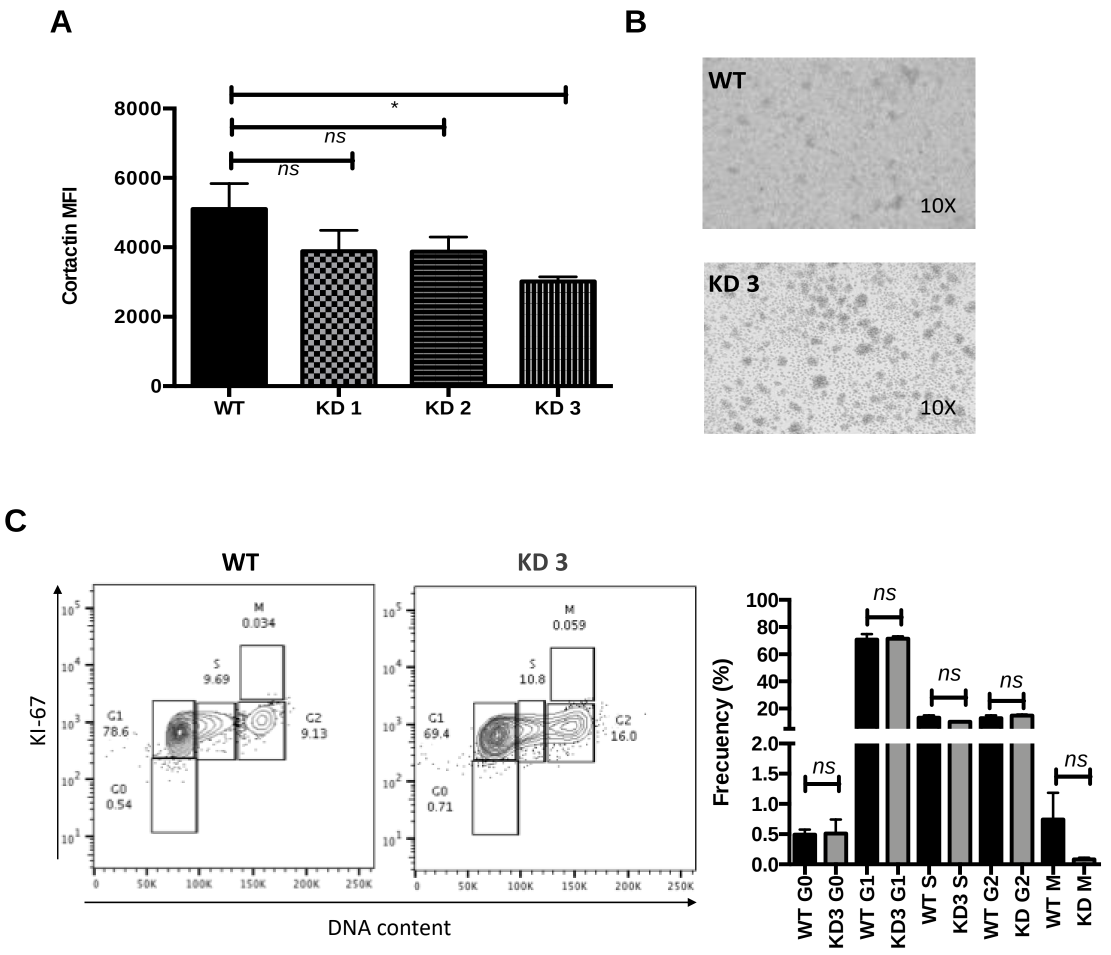

Characterization of stable cortactin knock-down (KD) REH cells.
Cortactin KD REH cells were generated by using three different CRISPR/Cas9 vectors. **A)** Cortactin expression levels were investigated by flow cytometry. Data are displayed as mean fluorescence intensity (MFI). While all vectors reduced cortactin expression, only the reduction in the KD 3 cells was significant. **B)** Bright field images of WT and KD REH cells are shown. **C)** The cell cycle status of WT and cortactin KD REH cells was investigated by flow cytometry with KI-67 and DAPI stainings. n=4, *<0.05; ns=not significant.

Supplemental Figure 2

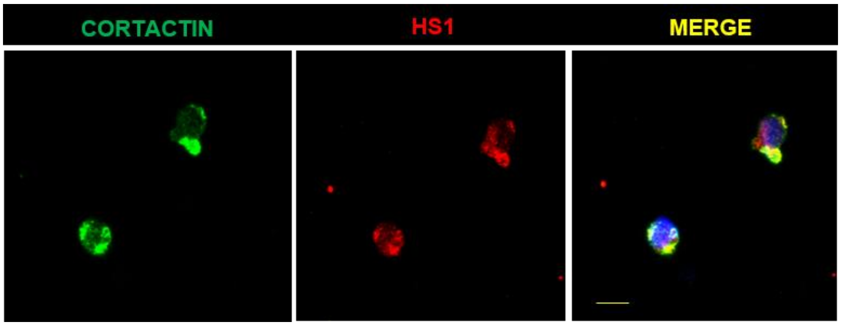

Cortactin and HS1 partially co-localize in REH cells.
Immunofluorescence staining of cortactin (green) and HS1 (red) in REH cells. Nuclei were stained using DAPI. Bar=20µm (n=3).

Supplemental Figure 3

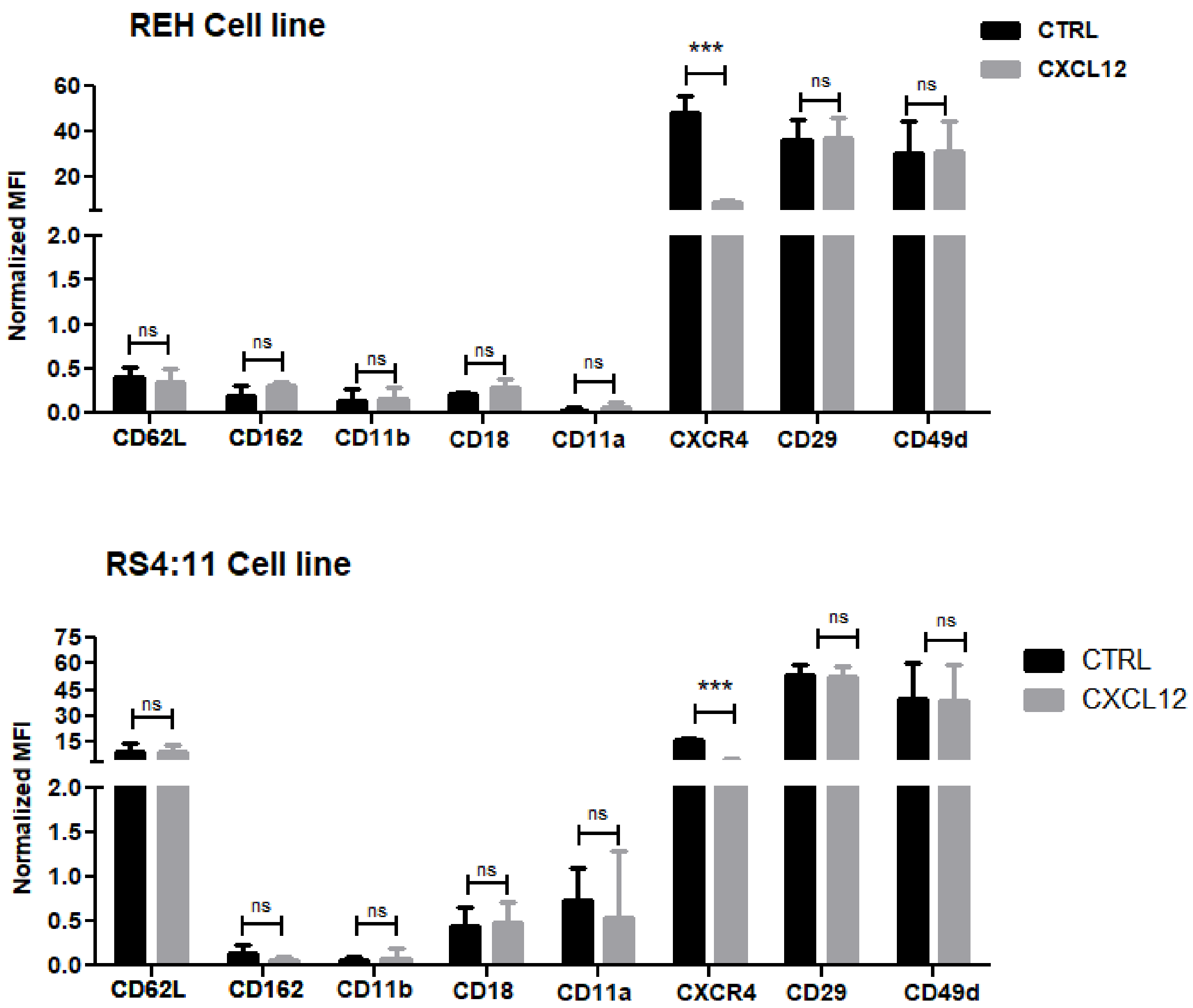

Expression of surface receptors in B-ALL cell lines.

Expression of L-Selectin (CD62L), CD162 (PSGL1), Mac-1 (CD11b/CD18), LFA-1 (CD11a/CD18), VLA-4 (CD49d/CD29) and CXCR4 were investigated in REH (top) and RS4:11 (bottom), with or without 100ng/ml CXCL12 for 4 h. Data are displayed as normalized mean fluorescence intensity (n=3). ns=non-significant; ***$p<0.001$.

Supplemental Figure 4

A

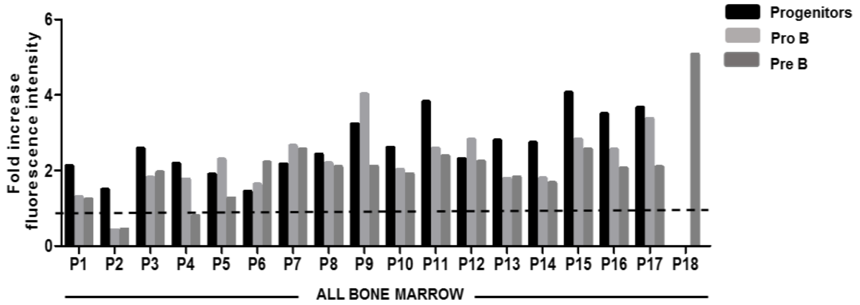

B

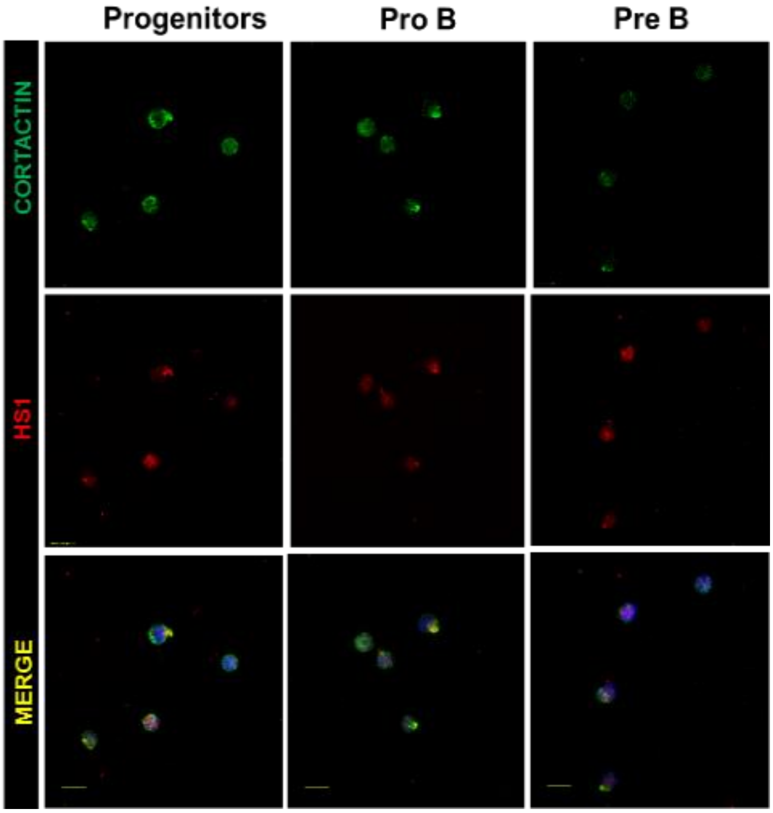

Cortactin expression levels in early leukemic populations from B-ALL patients.

A) Cortactin levels are heterogeneous in different B-cell precursors from B-ALL patients as determined by flow cytometry. Data are presented as fold increase of mean fluorescence intensity normalized to Umbilical cord blood mononuclear cells (dotted line). **B)** Representative immunofluorescence stainings of sorted BM precursors from B-ALL patients show that cortactin expression diminishes during maturation, whereas HS1 does not (n=3). Bar=20µm.

Supplemental Figure 5

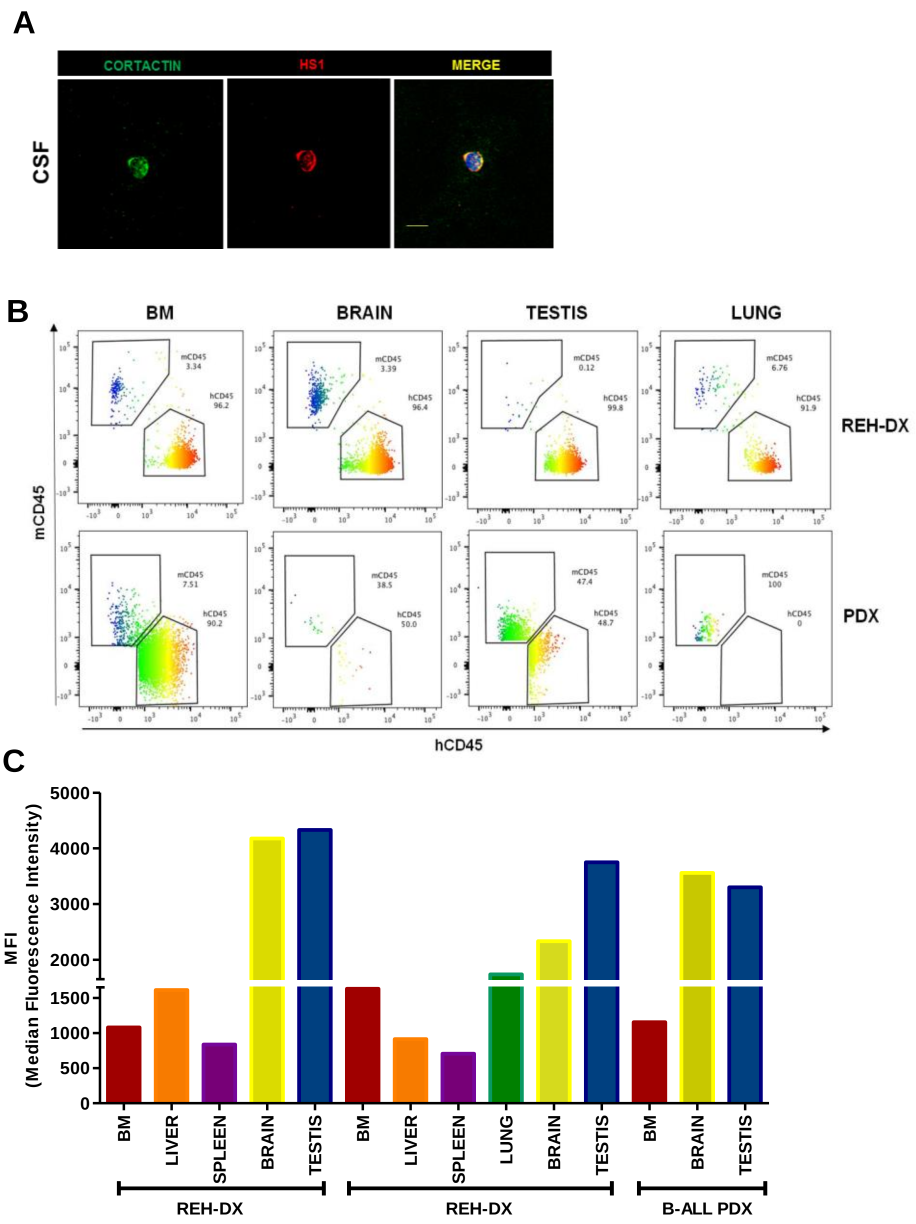

Infiltrated B-ALL cells express high levels of cortactin.

A) Representative image showing partial co-localization of cortactin (green) and HS1 (red) in B-ALL cells that infiltrated the cerebrospinal fluid. Bar=20µm. **B/C)** NSG mice were xeno-transplanted with REH or primary B-ALL cells. REH-derived xenografts (REH-DX) and patient-derived xenografts (PDX) were analyzed for infiltrated cells in BM, brain, testis and lungs by flow cytometry. **B)** Representative plots of gated live, infiltrated human leukocytes are shown for REH-DX and PDX (right panel). **C)** Single cortactin values of each xeno-transplant are depicted for the tested tissues.

Supplemental Figure 6

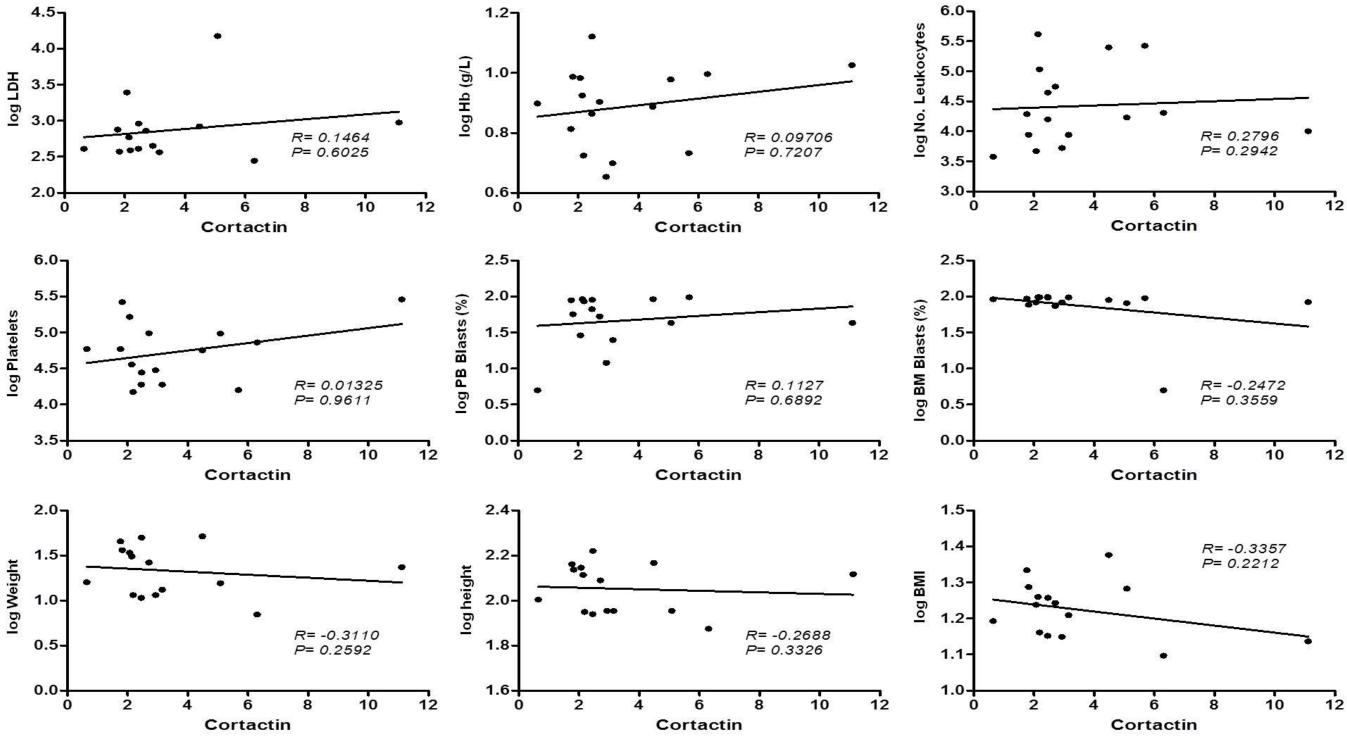

Correlation analysis of cortactin levels with patient data.

Fold increase expression of cortactin in B-ALL bone marrow cells (n=23) was determined by flow cytometry and correlated with the indicated patient data. Y-axis in logarithmic scale.

LDH = lactate-dehydrogenase, Hb= hemoglobin, PB= peripheral blood,

BM= bone marrow, BMI= body-mass index.

Supplemental Figure 7

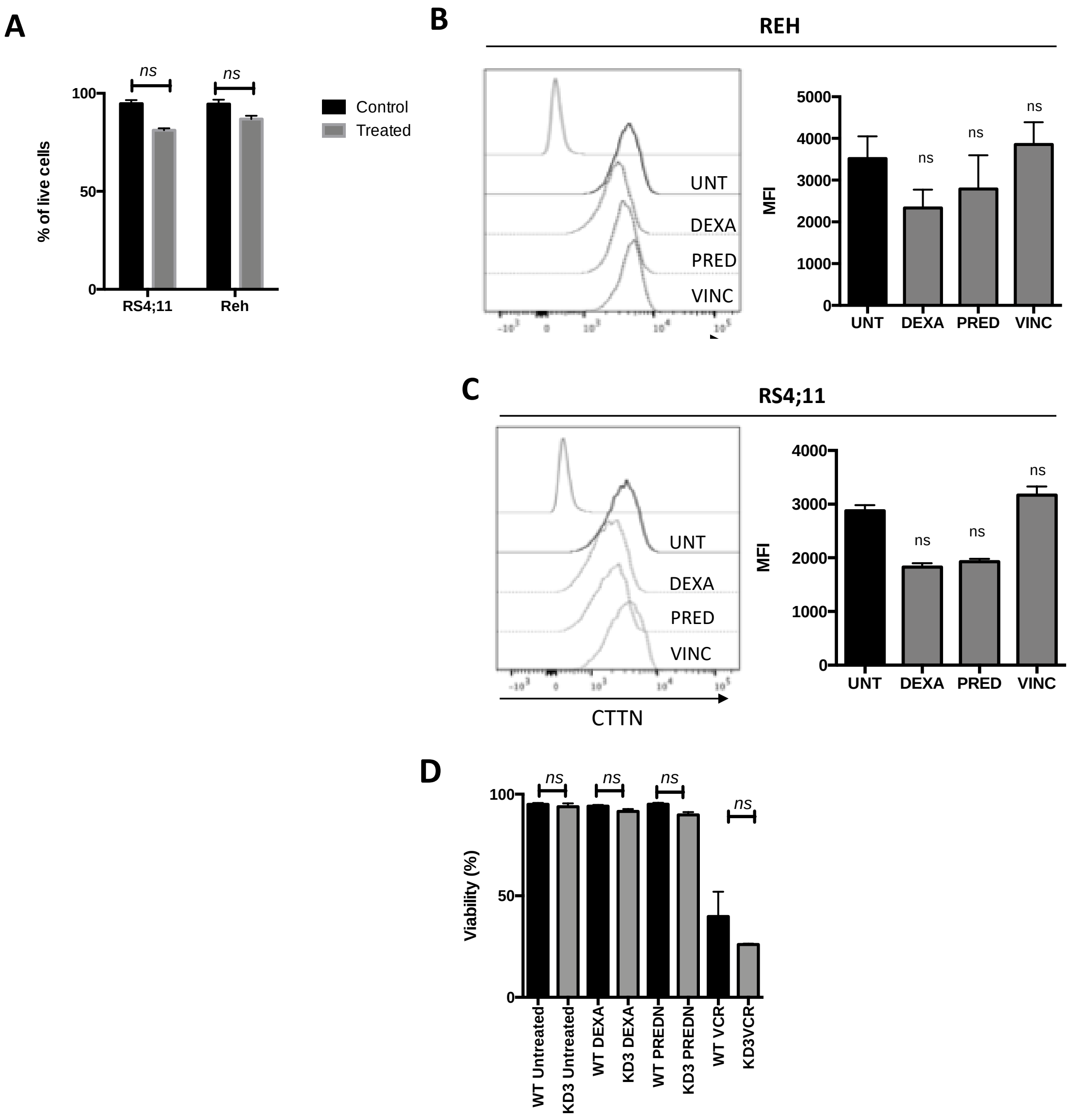

Cortactin expression is not critically affected after chemotherapy in B-ALL cells.
B-ALL cell lines RS4;11 and REH were treated with 5nM dexamethasone, 5nM prednisolone and 50ng/mL vincristine alone or in combination, and viability was assessed after 24 hours of treatment (**A**). Individual treatments were performed and cortactin levels were determined in surviving REH **(B),** or RS4:11 cells **(C)** 24 h after treatment. **D)** Viability after 48 hours of treatment was determined in stable REH cortactin-KD cells compared to control cells. n=5; ns=not significant.

Supplemental Figure 8

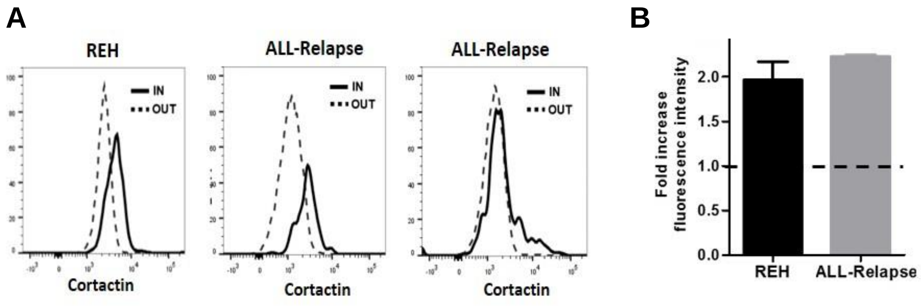

Cortactin levels correlate with bone marrow colonisation potential of relapsed B-ALL cells.
A) Cortactin levels in REH and primary relapsed B-ALL cells from two different patients were quantified from 3D-BM stromal spheroid co-cultures. Plots of $hCD45^{+}$-cells spheroid-colonising cells (IN) and in spheroid-surrounding cells (OUT) are shown. **B)** Colonizing cells expressed higher cortactin levels when normalized to non-colonizing $hCD45^{+}$-cells (set to 1).

Supplemental Figure 9

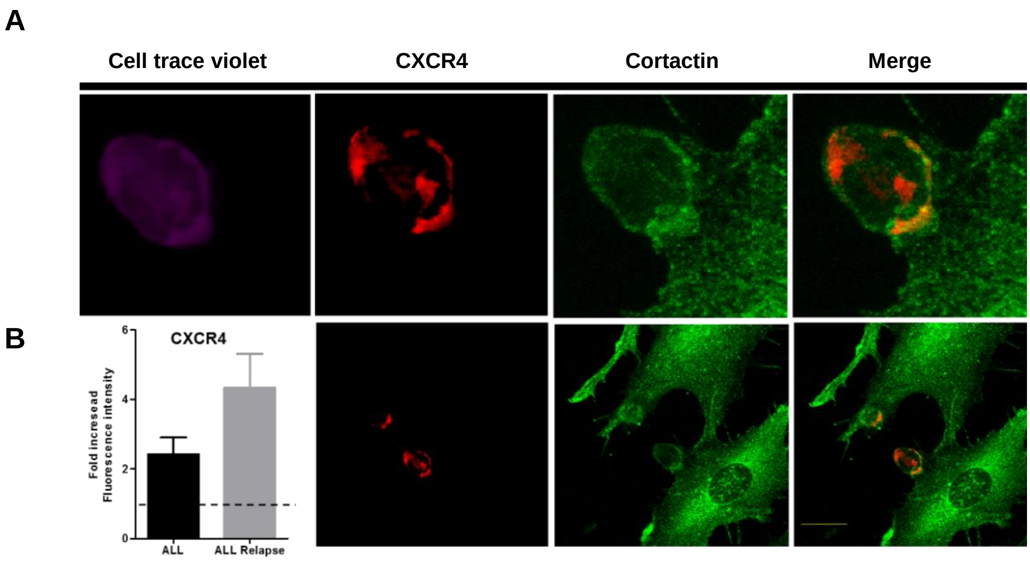

CXCR4 localizes in proximity to cortactin at cell contacts of B-ALL and stromal cells, and is upregulated after relapse.

A) CXCR4 (red) and cortactin (green) co-staining in co-cultures of REH and stromal cells. Bar=20µm.
B) CXCR4 expression was increased in relapsed BM samples. Data are normalized to CXCR4 in umbilical cord blood mononuclear cells (dotted line).

Supplemental Figure 10

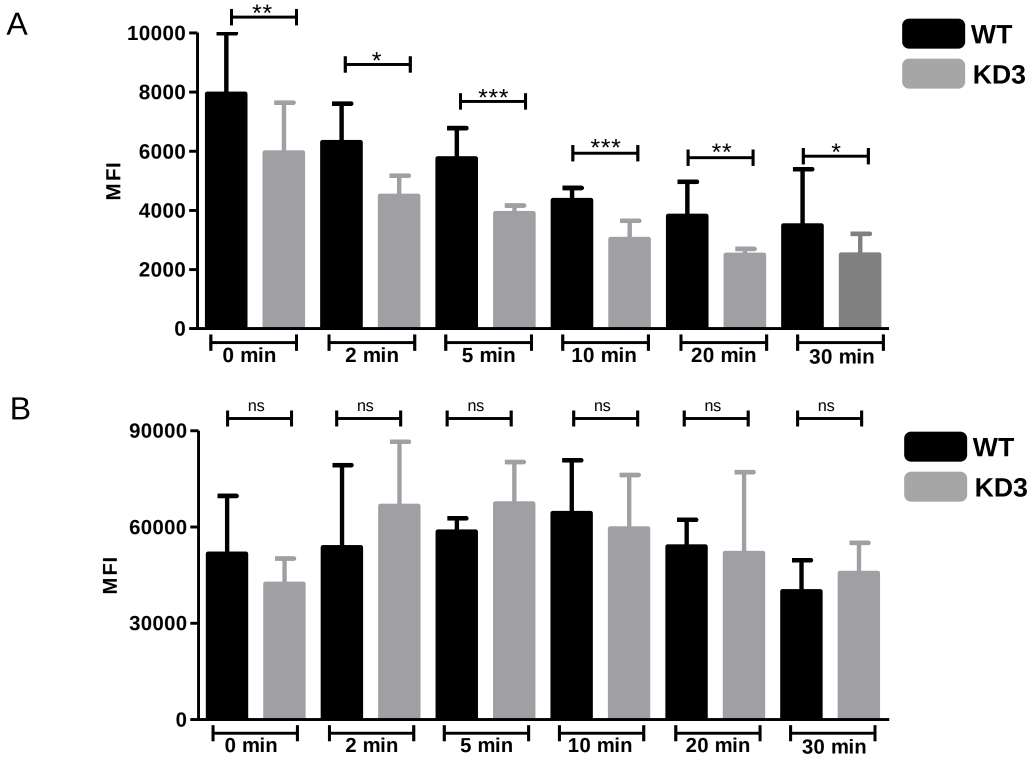

CXCR4 surface levels are lower in cortactin-depleted REH cells (KD3), but internalization occurs at similar levels.

A) Surface expression of CXCR4 was analysed by flow cytometry in non-permeabilised, untreated or CXCL12-treated KD3 and WT cells. **B)** Total CXCR4 levels were determined in permeabilized cells. n=3; *p<0.05;**p<0.01;***p<0.001; ns= not signifcant.

Supplemental Figure 11

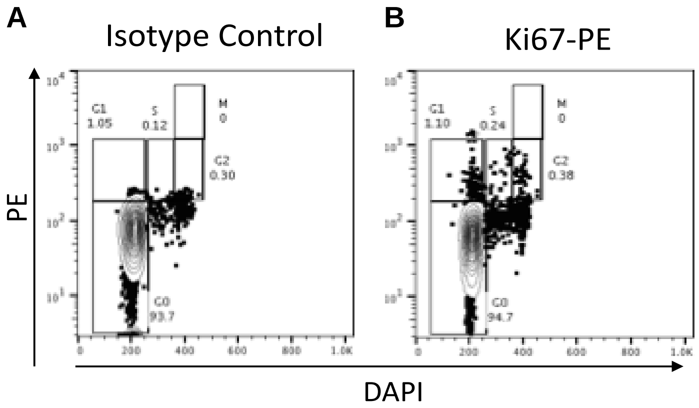

Cortactin^{high}-B-ALL cells that colonise the BM are in cell-cycle (compare Figure 6). A) Isotype controls of the experiment analysing cortactin levels in quiescent (G0) and cycling (G1-S-G2-M) cells as shown in Figure 6. **B)** NSG mice were xeno-transplanted with REH cells. A representative plot of hCD45^{+}-cells isolated from BM after establishment of the disease is shown (n=3).

Supplemental Figure 12

A

B

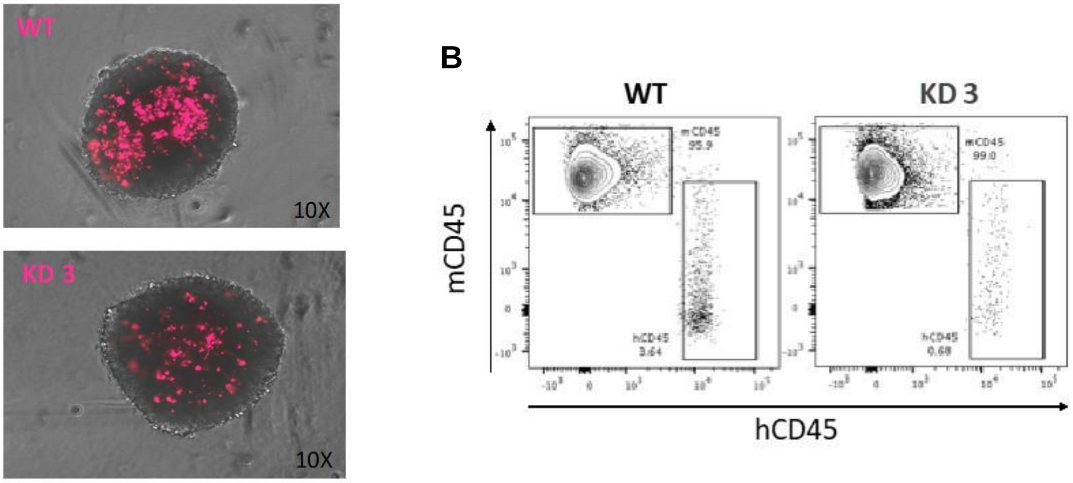

Cortactin is required for bone marrow engraftment and organ infiltration (compare Figure 7).
A) Stromal spheroids were co-cultured with labeled WT or KD3 REH cells and composition of spheroids after 24 hours was determined by fluorescence microscopy. **B)** Xenotransplantation assays were performed by i.v. injection of WT or KD3 REH cells into NSG mice. Overall engraftment by leukemic cells was investigated by flow cytometry (hCD45 vs mCD45 staining). n=3 for all experiments.

Supplemental Figure 13

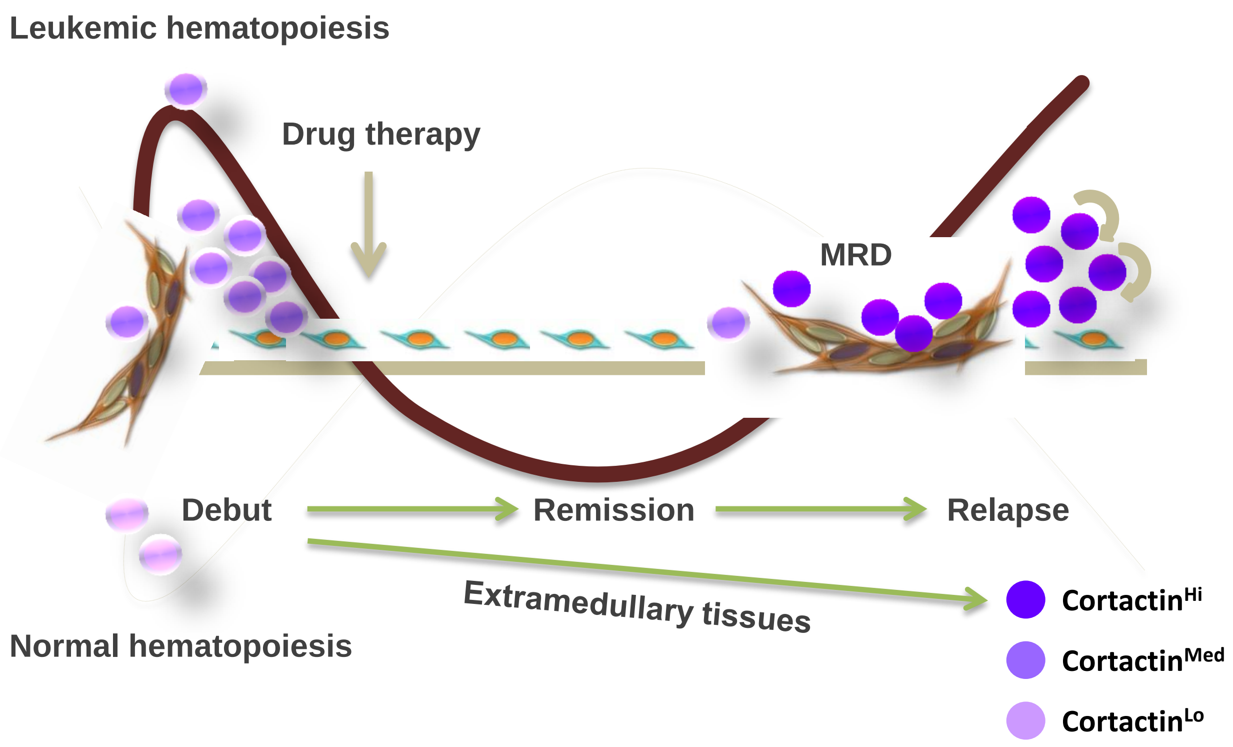

Working model.
The SV2 variant of cortactin is involved at debut in migration capabilities of early leukemic cells. During extramedullary infiltration and at relapse, cortactin expression is highest in cells endowed with niche-positioning and cycling properties.

Supplemental Table 1. Patient characteristics. BM=bone marrow; m=months; y=years; ND=not determined; PB=peripheral blood; Std=standard; BMI=body mass index; HB: hemoglobin; LDH= lactate dehydrogenase; CNS=central nervous system.

PATIENT	AGE	SEX	RISK	PHENOTYPE	HYPERLEU-COCYTOSIS	LEUKOCYTES ($X10^3$)	BLASTS (PB), %	BLASTS (BM), %	CHROMOSOMAL ABERRATIONS
**P1**	ND	Female	ND	Pro B	ND	ND	ND	ND	ND
**P2**	4y1m	Female	Std	Pro B	Negative	3.8	5	92	Negative
**P3**	ND	Female	ND	Pro/Pre B	ND	ND	ND	ND	ND
**P4**	8y	Female	ND	Pro B	ND	82.4	ND	ND	ND
**P5**	10y	Female	High	Pro B	Negative	8.8	57	77	Negative
**P6**	13y	Male	High	Pre B	Negative	19.4	89	93.7	Negative
**P7**	14y	Male	High	Pro B	Negative	44.3	90	96.7	Negative
**P8**	ND	Male	ND	Pro B	ND	ND	ND	ND	ND
**P9**	ND	Male	ND	Pre B	ND	ND	ND	ND	ND
**P10**	2y5m	Female	High	Pro/Pre B	Positive	108	86	7.6	Negative
**P11**	2y	Male	High	Pro B	Negative	5.3	12	83	**12:21**
**P12**	2y	Male	Std	Pro B	Negative	15.9	67	98.7	Negative
**P13**	7y	Male	High	Pro B	Positive	415	92	98.5	**4:11**
**P14**	11y	Male	High	Pre B	Negative	165	29	83	**12:21**
**P15**	3y	Female	High	Pro B	Negative	8.8	25	97.2	**12:21**
**P16**	8y	Female	High	Pre B	Positive	55.8	53	74	Negative
**P17**	ND	Male	ND	Pre B	ND	ND	ND	ND	ND
**P18**	2y	Male	High	Pre B	Negative	17	43	81	**1:19**
**P19**	3y	Female	High	Pro/PreB	Negative	73	0	5	Negative
**P20**	10	Male	High	Pro B	Positive	267	98	95	**9:22**
**P21**	8a 5m	Male	High	Pro B	Negative	10.1	43	84	Negative
**P22**	11y	Female	High	ProB	Negative	570	92	89.7	Negative
**P23**	ND	Male	ND	Pro B	ND	ND	ND	ND	ND

Supplemental Table 1 continued. Patient characteristics. BM=bone marrow; m=months; y=years; ND=not determined; PB=peripheral blood; Std=standard; BMI=body mass index; HB: hemoglobin; LDH= lactate dehydrogenase; CNS=central nervous system.

PATIENT	RESPONSE TO STEROIDS	BM REMISSION (DAY)	INFECTIONS DURING INDUCTION	HB (G/DL)	LDH (U/L)	PLATELETS	CYTOPENIA	HEPATOMEGALY	SPLENOMEGALY
**P1**	ND	ND	ND	ND	ND	ND	ND	ND	ND
**P2**	Yes	21	No	7.9	408	5900	No	No	No
**P3**	ND	ND	ND	ND	ND	ND	ND	ND	ND
**P4**	ND	ND	ND	ND	ND	ND	ND	ND	ND
**P5**	Yes	7	No	9.7	374	265000	No	No	No
**P6**	Yes	14	Yes	6.5	756	59000	Yes	Yes	Yes
**P7**	Yes	21	No	13.2	917	28000	No	No	No
**P8**	ND	ND	ND	ND	ND	ND	ND	ND	ND
**P9**	ND	ND	ND	ND	ND	ND	ND	ND	ND
**P10**	Yes	ND	Yes	5.3	390	15000	Yes	Yes	Yes
**P11**	No	7	No	4.5	449	30000	Yes	No	No
**P12**	Yes	14	No	7.3	409	19000	Yes	Yes	No
**P13**	Yes	14	No	8.4	591	36000	Yes	Yes	No
**P14**	Yes	7	No	9.6	2471	165000	Yes	Yes	No
**P15**	Yes	7	No	5	366	19000	Yes	No	No
**P16**	Yes	7	No	8	726	98000	No	Yes	No
**P17**	ND	ND	ND	ND	ND	ND	ND	ND	ND
**P18**	Yes	14	Yes	9.5	14970	97000	Yes	Yes	No
**P19**	Yes	7	No	9.9	279	73000	Yes	Yes	Yes
**P20**	No	28	No	5.4	ND	16000	Yes	No	No
**P21**	No	14	No	10.6	947	290000	No	No	No
**P22**	Yes	14	Yes	7.7	837	57000	Yes	Yes	Yes
**P23**	ND	ND	ND	ND	ND	ND	ND	ND	ND

Supplemental Table 1 continued. Patient characteristics. BM=bone marrow; m=months; y=years; ND=not determined; PB=peripheral blood; Std=standard; BMI=body mass index; HB: hemoglobin; LDH= lactate dehydrogenase; CNS=central nervous system.

PATIENT	ADENOMEGALY	WEIGHT (KG)	HEIGHT (CM)	BMI	NUTRITIONAL STATUS	PRIMARY INFILTRATION TO CNS	CNS NEGATIVE INFILTRATION (DAY)	RELAPSE	DEATH
**P1**	ND	ND	ND	ND	ND	ND	NA	No	ND
**P2**	No	16	101	15.6	Normal	No	NA	No	No
**P3**	ND	ND	ND	ND	ND	ND	NA	No	ND
**P4**	ND	ND	ND	ND	ND	ND	NA	No	ND
**P5**	Yes	36.3	137	19.4	Overweight	No	NA	No	No
**P6**	No	45.5	145	21.6	Normal	No	NA	No	Yes
**P7**	Yes	50	166	18.1	Normal	No	NA	No	No
**P8**	ND	ND	ND	ND	ND	ND	NA	No	ND
**P9**	ND	ND	ND	ND	ND	ND	NA	No	ND
**P10**	Yes	11.5	89	14.5	Malnutrition	No	NA	No	Yes
**P11**	Yes	11.5	90	14.1	Malnutrition	No	NA	No	No
**P12**	Yes	10.7	87	14.2	Normal	No	NA	No	NO
**P13**	Yes	30.8	130	18.2	Normal	No	NA	No	No
**P14**	No	34	140	17.3	Normal	No	NA	No	No
**P15**	Yes	13.2	90	16.2	Normal	No	NA	No	No
**P16**	Yes	26.5	123	17.5	Normal	Yes	10	No	No
**P17**	ND	ND	ND	ND	ND	ND	NA	No	ND
**P18**	Yes	15.6	90	19.2	Obesity	Yes	28	No	Yes
**P19**	Yes	7	75	12.5	Malnutrition	No	279	Yes	No
**P20**	Yes	ND	ND	ND	ND	No	NA	Yes	Yes
**P21**	Yes	23.5	131	13.7	Malnutrition	No	947	Yes	No
**P22**	Yes	51.5	147	23.8	Normal	No	837	Yes	No
**P23**	ND	ND	ND	ND	ND	ND	ND	Yes	ND
